# Degradation of Ibuprofen in flow-through system by the Electro-Fenton Process activated by two iron sources

**DOI:** 10.21203/rs.3.rs-2608922/v1

**Published:** 2023-04-03

**Authors:** Yuwei Zhao, Jiaxin Cui, Stephanie Sarrouf, Shayan Hojabri, Akram N. Alshawabkeh

**Affiliations:** a Department of Civil and Environmental Engineering, Northeastern University, Boston, 02115, MA, USA; b Changjiang Survey, Planning, Design and Research Co., Ltd., Wuhan 430010, Hubei, China

**Keywords:** Electro-Fenton, Flow-through system, Ibuprofen

## Abstract

The electrochemical degradation of ibuprofen (IBP) by electro-Fenton process has been studied in a flow-through system by evaluating the performance of two different iron sources, sacrificial cast iron anode and FeSO_4_ salt. The effect of operating conditions, including initial IBP concentration, cast iron anode location, initial FeSO_4_ concentration, applied current, the split current on the iron anode, solution pH, and flow rate on the efficacy of the process was evaluated. The sequence of the electrodes significantly influences ibuprofen removal. When using cast iron anode as iron source, placing the iron anode upstream achieved the best IBP removal rate. Split current of 3 mA applied on the iron anode out of 120 mA total current is the optimum current for remove 1 mg/L of IBP under a flow rate of 3 mL/min. There is a linear correlation between the applied current and the Fe^2+^ concentration in the FeSO_4_-system. The initial IBP concentration does not influence the rate of Fenton reaction. Flow rate influences the degradation efficiency as high flow rate dilutes the concentration of OH radicals in the electrolyte. FeSO_4_-system was less affected by the flow rate compared to the iron anode-system as the concentration of the Fe^2+^ was steady and not diluted by the flow rate. Both systems prefer acidic operation conditions than neutral and alkaline conditions. Iron-anode can be used as an external Fe^2+^ supply for the treatment for iron-free. These findings contribute in several ways to our understanding of the electro-Fenton process under flow conditions and provide a basis for how to design the reactor for the water treatment.

## 1. Introduction

Electrochemical advanced oxidation processes (EAOPs) have been widely developed for environmental remediation, especially for aqueous streams(Brillas, Sirés, and Oturan 2009). These processes electrogenerate highly reactive species, such as hydroxyl radicals, which can effectively break down aqueous pollutants such as toxic and persistent pesticides, organic synthesis dyes, pharmaceuticals and personal care products (PPCPs), and industrial pollutants(H. Zhang and Lemley 2006)(Miodrag Belosevic 2014)(W. Zhou, Meng, et al. 2019)(Yuan, Gou, et al. 2013)(W. Zhou, Rajic, et al. 2019). The Electro-Fenton (EF) process is one of the most studied EAO technologies. In the EF process, H_2_O_2_ is continuously generated at a suitable cathode fed with O_2_ or air (Equation 1). Once generated, along with the Fe^2+^ at acidic electrolyte, H_2_O_2_ is catalyzed to OH radicals via the Fenton process (Equation 2), and then Fe^2+^ is regenerated at the cathode (Equation 3). Compared to the traditional Fenton process, the EF can (1) produce H_2_O_2_ on-site, which avoids the risks of transport, storage, and handling of H_2_O_2_; (2) generate and regenerate Fe^2+^ in-situ to increase the removal efficiency and decrease the sludge production; (3) control the degradation kinetics to allow the mechanistic study(Brillas et al. 2009).


Equation 1 $$O_{2}+2H^{+}+2e^{-}\to H_{2}O_{2}$$

Equation 2 $$H_{2}O_{2}+H^{+}+Fe^{2+}\to Fe^{3+}+\cdot OH+H_{2}O$$

Equation 3 $$Fe^{3+}+e^{-}\to Fe^{2+}$$

The degradation efficiency of EF process can be influenced by several parameters such as O_2_ supply, stirring rate or liquid flow rate, temperature, pH, applied potential or current, nature and initial concentration of organics, and concentration of iron catalyst(M. Zhou et al. 2007)(X. Zhang et al. 2009)(Su et al. 2019)(F. Yu et al. 2014). The influencing parameters can be separated into two categories. One affects the degradation rate by directly influencing the generation of hydroxyl radicals, and the other influences H_2_O_2_ production to affect the OH radicals production indirectly.

The pH of the electrolyte is a critical factor for maintaining the efficiency of the EF process. The optimal pH range for the Electro-Fenton reaction is typically around 3(X. Yu et al. 2015)(Haber et al. 1934). At this pH range, ferrous iron (Fe^2+^) is in its highest oxidation state, which is necessary for the generation of hydroxyl radicals. Operating under unexpected pH values can cause lower OH radical generation and more iron sludge production. For example, previous research showed that the optimum of the pH is 3 on the removal of methyl parathion with 0.1 mM Fe^3+^ by carbon-felt. The mineralization rate is decreasing at pH 4, where part of Fe^3+^ forms Fe(OH)_3_ precipitation, and at pH 1, where Fe^2+^ complexes with H_2_O_2_ and other ions, such as SO_4_^2−^ (Festos, n.d.). Soluble iron salts are usually used as the source of iron ions in electrochemical system design. The catalytical behavior of Fe^2+^/Fe^3+^ couple considering the cathodic regeneration of Fe2+ mainly depends on the cathode material(Brillas and Casado 2002)(Brillas et al. 2009). For example, Fe^2+^ is the preferred catalyst for gas diffusion electrode at the early stage of the EF process (Sirés et al. 2007). Conversely, Fe^3+^ and Fe^2+^ ions have similar behavior on degradation of malachite green due to the fast Fe^2+^ regeneration rate on carbon felt(Oturan et al. 2008). The alternative approach is to add a sacrificial iron anode, which can continuously release the Fe^2+^ to the electrolyte (Cretin and Oturan 2016). The ferrous iron production can be controlled by adjusting the applied current. This method overcomes the handle and use of commercial ferrous salt and avoids some unexpected oxidation of Fe^2+^ during handling and transportation.

Cathode material and cell configuration are another two influencing factors. Carbon is the most popular cathode material for the 2e-ORR because of its high over potential for H2 evolution and low catalytic activity for H_2_O_2_ decomposition, as well as good stability, conductivity, and chemical resistance. However, due to the poor solubility of O_2_ (≈1×10^−3^ mol /dm^3^) in aqueous solutions(Yamanaka et al. 2003), traditional carbon materials can only produce a limited amount of H_2_O_2_ with low current efficiency. Different modification methods were developed to enhance the performance of carbon cathodes. Introducing different carbon materials, such as carbon black (F. Yu, Zhou, and Yu 2015)(Castañeda et al. 2017), carbon nanotubes (CNTs) (Khataee et al. 2011)(Gao et al. 2015)(Ai et al. 2008), graphene(Su et al. 2019)(Kim et al. 2018)(Le et al. 2015), and acetylene black(Sheng et al. 2011)(Liu et al. 2005), to commercially available carbon materials is an effective method. PTFE or PVDF usually works as a binder to connect active carbon materials with the support. Sheng et al. fabricated an acetylene black-PTFE electrode that achieves accumulation of H_2_O_2_ concentration of 677.5 mg/L with a production rate of 54.2 mg/(cm·h)(Sheng et al. 2014). The carbon-PTFE layer worked as diffusion layer and catalyst layer, forming a highly porous structure with more active sites on the surface, which can support a very good H_2_O_2_ production. Cell configuration, including undivided and divided cells, significantly affects electrogeneration of H_2_O_2_. Generally, the divided cell, with two compartments, is separated by glass grits, a membrane, or a diaphragm for the electrolysis, which prevent the mixing of cathodically produced H_2_O_2_ and anolyte(Lin et al. 2014)(G. Zhang, Zhou, and Yang 2015). This design avoids the decomposition of H_2_O_2_ at the anode. The undivided cell only has one compartment for the electrolysis(Yuan et al. 2006)(Mao, Ciblak, et al. 2012)(Yuan, Chen, et al. 2013)(Mao, Yuan, et al. 2012). It has lower cell voltage require for H_2_O_2_ generation but with H_2_O_2_ decomposition at the anode compared to the divided cell.

In previous research, we designed a flow-through system for H_2_O_2_ generation with electrodes vertically stacked in a flow column reactor(Zhao et al. 2020)(W. Zhou et al. 2018). Electrolyte flow upward through the anode first then through the cathode. H_2_O_2_ produced at the cathode is utilized for Electro-Fenton process. This design only has one compartment, and it can avoid the H_2_O_2_ decomposition at cathodes. We also proposed an modification method which added a PDMS dampproof coating layer at the PTFE covered graphite felt to enhance the longevity of the cathode (Zhao et al. 2022). This system showed a good performance on electrogeneration of H_2_O_2_ under flow conditions.

The goal of this research is to evaluate the performance of the Electro-Fenton process in the flow-through system that we validated in prior research. Two iron sources, FeSO_4_ salt and sacrificial iron anode, were evaluated and compared on the degradation of Ibuprofen, the model contaminant, under flow conditions. Different operating parameters were evaluated on the influence of EF process to achieve the optimum degradation rate.

## 2. Experiment

### 2.1 Materials

All chemicals used in this study are analytical grade. The chemicals used in this study include hydrogen peroxide (30%, Fisher Scientific), calcium sulfate (99%, Sigma-Aldrich), sodium sulfate (anhydrous, ≥99%, Sigma-Aldrich), sulfuric acid (Fisher Scientific), sodium hydroxide (Sigma-Aldrich), titanium sulfate (99.9%, Sigma-Aldrich), 4-isobutyl-alpha-methylphenylacetic acid (99% Alfa Aesar), ammonium acetate (97% min, Alfa Aesar), ferrous sulfate Heptahydrate (≥99%, Fisher Chemical), 1–10 phenanthroline monohydrate (≥ 99%, ACRO ORGANIC), hydroxylamine hydrochloride (>97%, ACRO ORGANIC). PTFE (60%), used as a binder and hydrophobic coating, is purchased from Sigma-Aldrich. PDMS is obtained from Dow Corning for the dampproof coating. Anode electrode is Titanium-mixed metal oxide (Ti/MMO), consisting of IrO_2_ and Ta_2_O_5_ coating on Titanium, with a diameter of 2.9 cm and 2 mm in thickness. The Ti/MMO electrodes were immersed into diluted H_2_SO_4_ solution (10% wt) for an hour to remove the heavy metal at the surface, then were rinsed with DI water several times before assembly. Cast iron anode (McMaster-Carr, USA) was cut into a diameter of 3.2 cm and thickness of 0.5cm. 7 holes with a diameter of 2 mm were evenly distributed on the electrode to allow the electrolyte pass through. Graphite felt (G100, Fuel Cell Store) was cut into pieces with a dimension of 4.3 cm diameters, which is the same as the column reactor’s inner diameter.

### 2.2 Cathode modification

Graphite felt (GF) was degreased in an ultrasonic bath with DI water for 8 hours, then dried at 80 °C for 12 hours. The pretreated GF was firstly coated with 0.5 mL of PTFE (60%) on it (Ref). Since the volume of PTFE to the area of GF electrode ratio (mL:cm^2^) is 1:29, the PTFE covered GF was marked as GF-(1:29). Then, the GF-(1:29) was used for the PDMS dampproof coating(Zhao et al. 2020). Since the mass of the PDMS is 20 and 50, the electrodes with PDMS coating were marked as GF-(20) and GF-(50), respectively.

### 2.3 Reactor design and experimental methods

**Figure 1:**
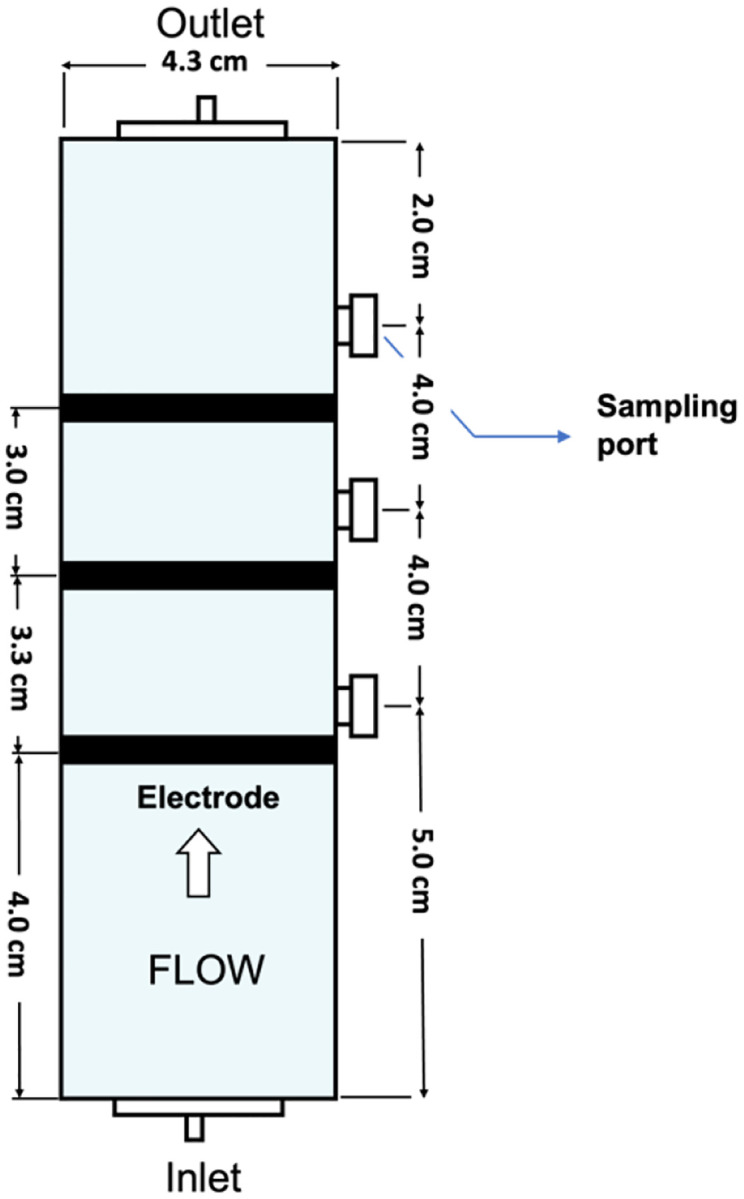
Design of the Reactor

A vertical acrylic column was used as an electrochemical flow-through reactor with an inner diameter of 4.3 cm and length of 15 cm, including three sample ports at 5 cm, 9 cm, and 13 cm from the bottom of the column. Electrodes were placed in parallel with respect to the flow direction at 4 cm, 7.7 cm, and 10.3 cm from the bottom of the column (Figure 1). Titanium rods were fabricated at electrode levels at electrical connections to hold the electrodes in place and convey electrical current.

**Figure 2:**
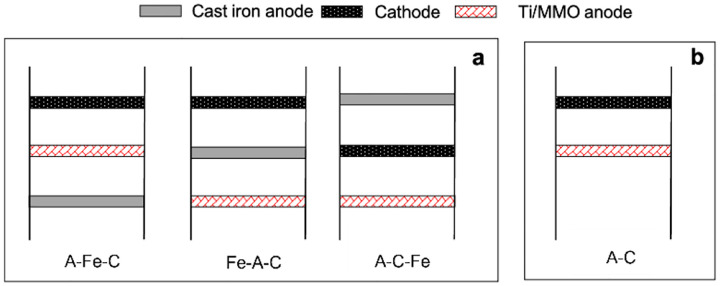
Sequences of the electrodes for a) Cast iron anode system; b) FeSO_4_ system

Two sets of experiments were conducted in this research. One uses cast iron anode as the iron source, and the other directly add FeSO_4_ chemical in the electrolyte as the iron sources for the degradation experiments. All experiments were conducted in the same reactor. Experiments with cast iron anode were tested under different combinations of electrode sequences (Figure 2a). The sequences (from bottom to top) of cast iron-Ti/MMO-GF, Ti/MMO-cast iron-GF, and Ti/MMO-GF-cast iron were marked as Fe-A-C, A-Fe-C, and A-C-Fe, respectively. Experiments with FeSO_4_ as the iron source only contain two electrodes: Ti/MMO anode (at the bottom) and GF cathode (at the top) (Figure 2b).

All experiments were using stimulated groundwater as the electrolyte (0.5 mg/L of CaSO_4_ and 3 mg/L of Na_2_SO_4_) with different concentrations of IBP in it. Different FeSO_4_ was also added for the experiment without iron anode. A control experiment was conducted without any iron source to test the removal of IBP by H_2_O_2_ and adsorption.

### 2.4 Analytical Method

The iron concentration was determined by the 1–10 phenanthroline analytical method(Komadel and Stucki 1988). For measuring the total dissolved iron, a 0.5 mL sample taken from the sampling ports were filtered by the 0.45 μm syringe filter, then mixed with 0.25 mL of 10% of hydroxylamine hydrochloride to reduce all dissolved iron to ferrous iron. 1 mL of the acetic acid buffer (20 g acetic ammonia and 25 mL acetic acid in 100 mL of water) was used to adjust the pH to 3 to 5, and 1 mL of the 1–10 phenanthroline monohydrate (1 g/L) was added subsequently to the sample. The solution was measured at 510 nm wavelength using a UV-VIS spectrophotometer (SHIMADZU UV-1800). The concentration of ferrous iron was measured with the same method without adding the hydroxylamine hydrochloride. The pH was measured by a pH meter (Thermo Scientific).

Ibuprofen was measured by 1200 Infinity Series HPLC (Agilent) equipped with a 1260 diode array detector (DAD), a 1260 fluorescence detector (FLD), and an Agilent Eclipse AAA C18 column (4.6×150mm). The mobile phase was a mixture of methanol and water (70:30, v/v) at a flow rate of 1 mL/min. The detection wavelengths for DAD were set at 282 nm, and the column temperature at 40°C(Yuan, Gou, et al. 2013).

The removal of IBP in the flow-through system approximately followed the pseudo-first-order reaction at the beginning of the experiment. The model is given by:

Equation 4 $$\text{ln}\left(\frac{C}{C_{0}}\right)=kt$$

Where t is the reaction time (min), k is the rate constant (/min), and C_0_ and C are the concentration of IBP (mg/L) at times of t=0 and t=t, respectively.

The total removed IBP (mg) was estimated by the following equation:

Equation 5 $$\text{Total removal IBP}=v\int_{t_{0}}^{t}\left(C_{i}-C\right)dt$$

Where t is the reaction time (min), Ci is the initial concentration of IBP (mg/L) and C is the concentration at t.

The charge efficiency (mg/C) was calculated by:

Equation 6 $$\text{Charge Efficiency}=\frac{\text{Total removed IBP}}{\text{It}}$$

Where t is the reaction time (s), and I is the applied current (A).

## 3. Results and Discussion

### 3.1 Degradation of ibuprofen by iron anode as the iron source

#### 3.1.1 Effect of different modified cathodes

**Figure 3:**
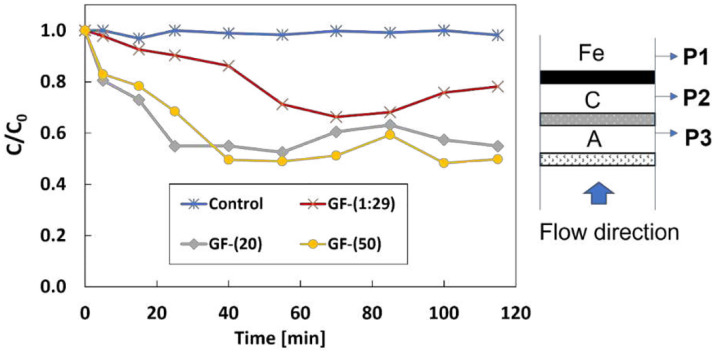
Effect of different modified cathodes on the removal of 1 mg/L of IBP under 120 mA and 3 mL/min

Figure 3 shows ibuprofen removal using two modification methods under the flow rate of 3 mL/min and the total current of 120 mA. Of total current, 3 mA of the current was split to the cast iron anode to generate Fe^2+^ in the electrolyte. The sequence of the electrode is A-C-Fe from bottom to top (along flow direction). One control experiment was conducted under the same conditions but without a sacrificial iron anode. Thus no OH radicals were generated in the system.

The removal rate of IBP is near zero in the control experiment, indicating that ibuprofen is unable to be oxidized by H_2_O_2_ or Ti-MMO anode. The removal rate for the GF-(1:29), the GF-(20), and the GF-(50) is 22%, 45.1%, and 50.2%, respectively. In the case of the GF-(1:29) cathode, a large oxygen bubble appeared at the bottom of the GF-(1:29) cathode after 1 hour due to the electrowetting, indicating that the gaseous oxygen transport rate dropped below the generation rate. The mass transfer in the system decreased leading to a lower degradation efficiency. Due to the dampproof coating, the electrowetting did not happen on the GF-(20) and GF-(50) in 115 mins. Thus, the cathode with the dampproof coating had better performance on IBP degradation than the one without the coating. Cathodes with different amounts of PMDS have very similar performance for degradation. Thus, the PDMS content does not affect the removal rate in the short term. All the following experiments were conducted with the GF-(20) since it used a small amount of PDMS.

#### 3.1.2 Effect of the location of the cast iron anode

The cast iron location can affect the production of OH radicals by influencing dissolved iron concentration at the cathode. Experiments were operated under the sequence of:

1. Anode-Iron Anode-Cathode (A-Fe-C).
2. Anode-Cathode-Iron Anode (A-C-Fe).
3. Iron Anode-Anode-Cathode (Fe-A-C)

**Figure 4:**
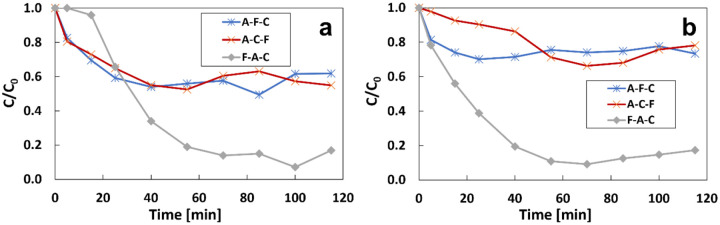
Effect of the location of the iron anode with the spilt current of a) 3 mA and b) 10 mA under 3 mL/min, the total current of 120 mA, and initial IBP of 1 mg/L

From bottom to top with a split current of 3 mA (Figure 4a) or 10 mA (Figure 4b) and the total current of 120 mA.

The removal rate at steady state was 38.2% and 45.1% when the iron anode was at the middle and the top. When the iron anode was located at the bottom of the electrode sequence, the removal efficiency was 83.2% at 115 mins, the highest result among the three experiments. Experiments were also conducted under 10 mA of split current on the sacrificial iron anode. A similar trend was observed in Figure 4b: A-Fe-C and A-C-Fe have very low removal rates of 27% and 22%, compared to 82.8% for Fe-A-C at 115 mins.

**Figure 5:**
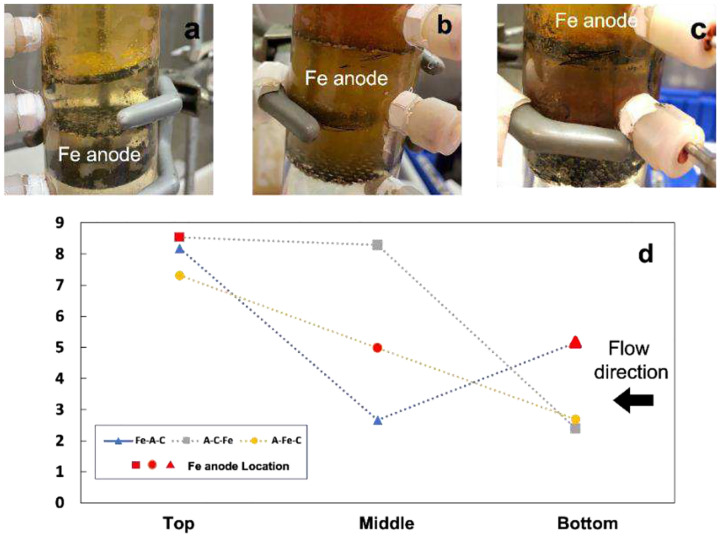
Photos of the reactor with the sequence of a) Fe-A-C, b) A-Fe-C, c) A-C-Fe and the split current of 10 mA; d) pH value from different sampling ports at 60 mins with the split current of 10 mA

Figure 5d shows the pH value from different sampling ports at 60 mins for experiments conducted under different iron anode locations. pH values at the Ti/MMO anode were acidic for all experiments because water electrolysis generates H^+^ at the anode through Equation 8. The sacrificial iron anode in experiments Fe-A-C and A-Fe-C was in an acidic environment, but for experiment A-C-Fe, it was in an alkaline environment.


Equation 7 $$H_{2}O\to 2H_{2}\left(g\right)+4H^{+}+4e^{-}$$

In experiment A-Fe-C, Fe^2+^ generated at the iron anode directly contacted the anodic oxygen produced on the Ti/MMO anode at pH 5. Thus, most of the Fe^2+^ would be oxidized to Fe^3+^, resulting in Fe(OH)_3_ precipitation (Figure 5b). Although the iron anode at the very top of the reactor (A-C-Fe) can avoid directly contacting the anodized oxygen, the pH at the Fe anode was alkaline, under which more Fe^3+^ would precipitate with OH^−^. Some of the Fe^3+^ precipitation also occurred on the cathode(Figure 5c), inhibiting the reactions at the cathode, including 2e-ORR. Thus, the concentration of Fe^2+^ is limited for the Fenton reaction in experiments A-Fe-C and A-C-Fe.

Although Fe^2+^ contacts the Ti/MMO anode in the experiment Fe-A-C with the electrolyte flow, no significant precipitation was observed. The low pH at Ti/MMO anode (Figure 5a) can decrease the oxidation rate of Fe^2+^ and inhibit the formation of iron precipitation. This observation is compatible with previous research that has shown retarded oxidation rates under very acidic conditions (Jones et al. 2014). More dissolved iron will be available for the Fenton reaction when this acidic electrolyte arrives at the cathode. These results explain why Fe-A-C has better IBP removal than the other experiments.

#### 3.1.3 Effect of the split current on the iron anode

**Figure 6:**
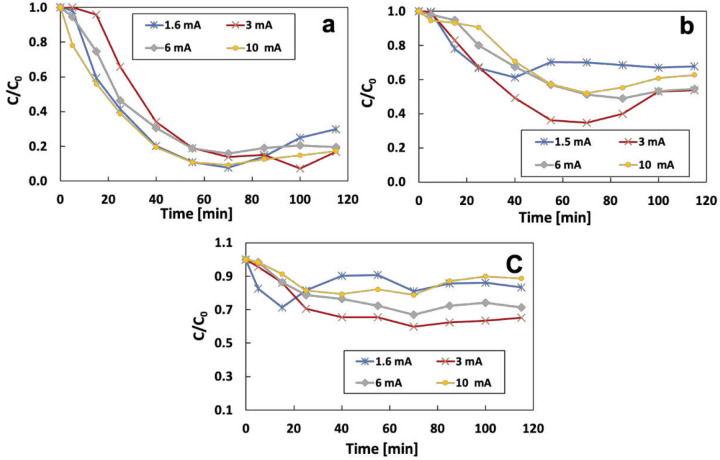
Effect of the split current on the removal of a) 1 mg/L, b) 3 mg/L, and c) 5 mg/L of IBP

Fe^2+^ as the catalyst for the Fenton reaction can directly affect the OH radical production. The generation of Fe^2+^ can be changed by adjusting the split current on the sacrificial iron anode in the electrochemical system. Experiments were conducted to analyze the removal rate of different initial IBP concentration (1, 3, and 5 mg/L) under different split currents (1.6, 3, 6, and 10 mA)(Figure 6). When the initial concentration of IBP was 1 mg/L, the ratio of IBP left in the electrolyte at 115 mins was 69.2%, 83.2%, 80.5%, and 82.8% for split current of 1.6, 3, 6, and 10 mA, respectively. Increasing initial IBP to 3 mg/L decreased the removal rate to 32.4%, 46.4%, 45.6%, and 37.4%. When the concentration of IBP was further increased to 5 mg/L, the removal rate was 26.7%, 34.9%, 28.6%, and 11.5%. The optimum removal rate was achieved at the split current of 3 mA for different initial IBP values.

**Figure 7:**
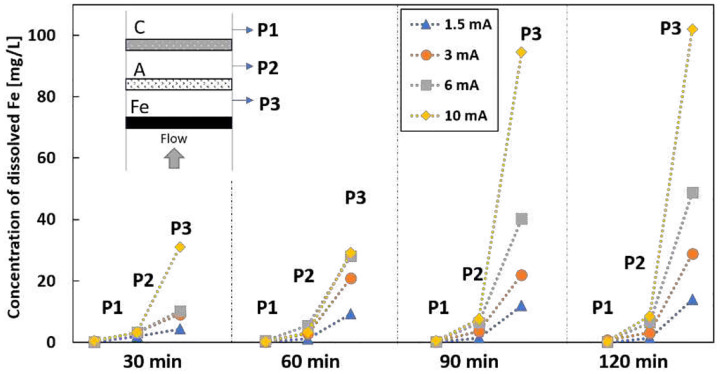
Concentration of dissolved iron in the electrolyte under different split current with initial IBP of 1 mg/L at different time

Figure 7 shows the dissolved iron concentration at different locations as a function of time and current. A higher split current generated more dissolved iron at sampling P3. There was a sharp decrease when the electrolyte passed the anode, as most dissolved iron will be oxidized to Fe^3+^ after passing the anode. P1 has the minimum amount of dissolved iron because the pH at the cathode is alkaline, leading to iron precipitation. Increasing dissolved iron in the electrolyte can enhance the Fenton reaction, but excess iron can consume OH radicals and generate more precipitation. Since the production of H_2_O_2_ should be similar under different split currents, the optimum removal rate appears under the maximum OH radical production. The concentration of initial IBP does not affect the generation of OH radicals. Thus, the optimum removal rate appears at 3 mA for different initial IBP.

#### 3.1.4 Effect of the total current applied on the system

**Figure 8:**
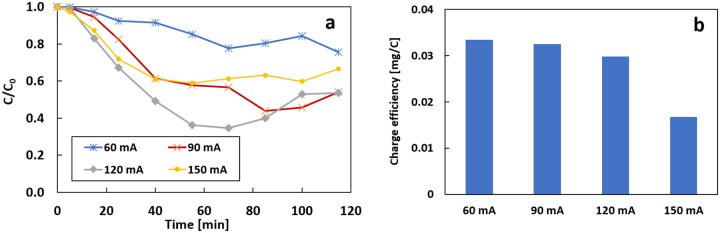
a) effect of the total current on the removal of 3 mg/L of IBP; b) the charge efficiency of different total current

Figure 8a shows the effect of total current on the removal of IBP in the flow-through system. Changing the total current can change the reaction rate on the cathode and the Ti/MMO anode. The removal rate was 24.5%, 46.1%, 46.6%, and 33.5% when the current was 60, 90, 120, and 150 mA, respectively. The best removal rate appeared at 120 mA with the highest k value (Table 1). The IBP removal was normalized by charge in Figure 8b. The charge efficiency was very similar at 60 mA and 90 mA (0.033 and 0.032 mg/C), then decreased to 0.029 and 0.017 mg/C when the current was increased to 120 mA and 150 mA. A similar trend was observed at the current efficiency of the electrogenenration of H_2_O_2_ in our previous research(Zhao et al. 2020).

Previous results showed that the yield of H_2_O_2_ was low at the beginning of the experiment and took time to stabilize at a low current. Hydrogen peroxide, as the precursor of the Fenton reaction, can directly affect the removal rate. This explains why there is a delay in the degradation experiments at 60 mA and 90 mA and why the experiment at 60 mA requires a longer time to reach the steady state (Table 1).

**Figure 9:**
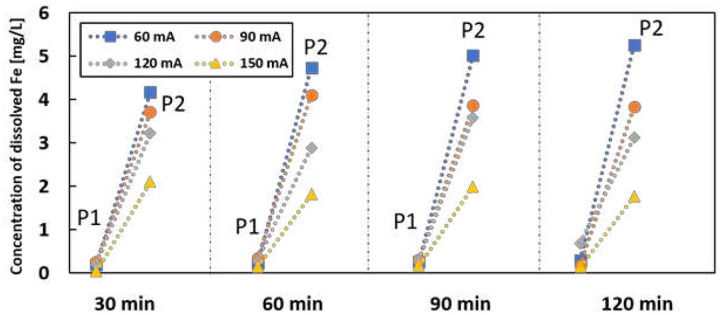
Concentration of dissolved iron from different sampling ports at different total applied current

The production of Fe^2+^ at the iron anode is the same when the total current varies because the split current is the same. Increasing current enhanced water electrolysis on the Ti/MMO anode and the 2e-ORR at the cathode. More H_2_O_2_ can be generated for the Fenton reaction. However, increasing anodic oxygen production can oxidize more Fe^2+^ to produce iron precipitation. Thus, the amount of dissolved iron at P2 that can be supplied to the Fenton reaction decreases with increasing current (Figure 9). Previous research also showed that the electrogeneration of H_2_O_2_ decreased when the current was over the optimum value because the decomposition reaction was enhanced. Thus, the optimum current is 120 mA to achieve the best removal rate.

#### 3.1.5 Effect of the flow rate

**Figure 10:**
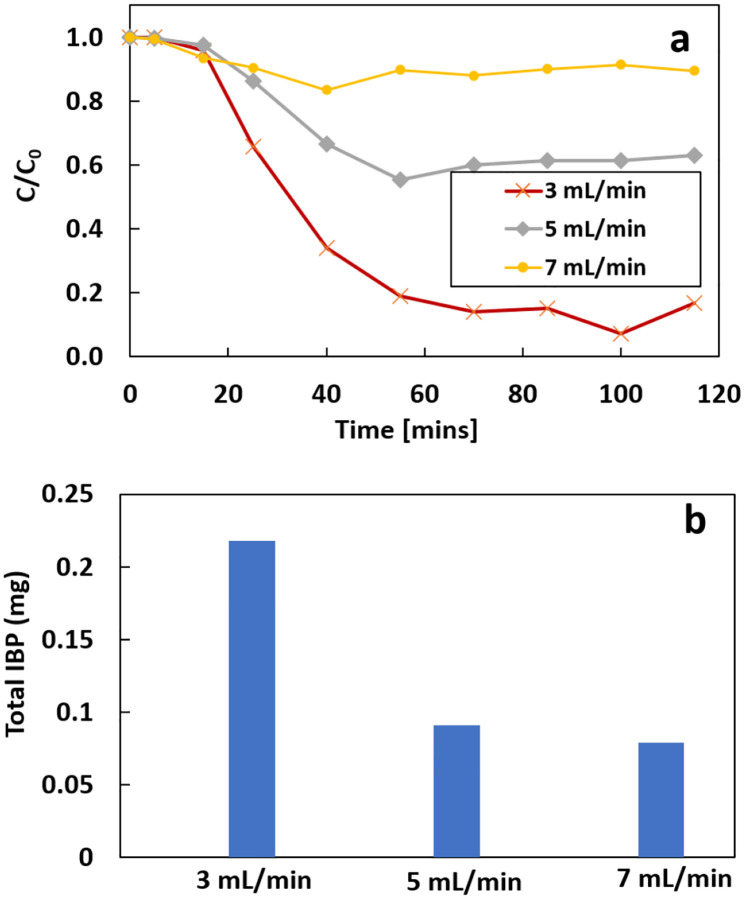
a) the removal rate and b) total removal IBP in 115 mins under different flow rate

Figure 10 shows the effect of the flow rate on the removal of 1 mg/L IBP. The ratio of IBP left in the electrolyte at 115 min is 16.9%, 63.1%, and 89.5% under the flow rates of 3, 5, and 7 mL/min, respectively. The removal rate decreased with the increase in flow rate. A similar trend was shown for the total removal of IBP under different flow rates. The total removal of IBP decreased from 0.22 mg to 0.09 mg and 0.08 mg when the flow rate increased from 3 to 5 and 7 mL/min, respectively. The higher flow rate has a lower k value and needs less time to reach the steady state (Table 2). Increasing the flow rate shortens the retention time for oxygen on the cathode, which can decrease the reaction rate for the 2e-ORR. A decrease in H_2_O_2_ concentration will lower the generation of OH radicals as well. Increasing the flow rate also dilutes the concentration of OH radicals in the electrolyte. Thus, the removal rate will decrease when the initial concentration of IBP remains constant.

**Figure 11:**
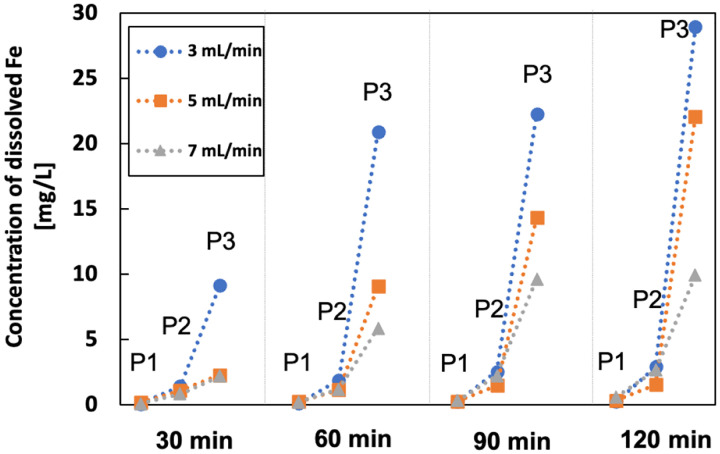
Concentration of dissolved iron at different flow rate

The production of Fe^2+^ on the iron anode remains the same since the current split is constant, but the increased flow rate dilutes the dissolved iron in the electrolyte, which decreases the concentration of the dissolved iron at P3 (Figure 11). However, the iron content at P2 remains similar, mainly because the concentration of anodic oxygen decreased as well with the flow increasing.

#### 3.1.6 Effect of the pH

**Figure 12:**
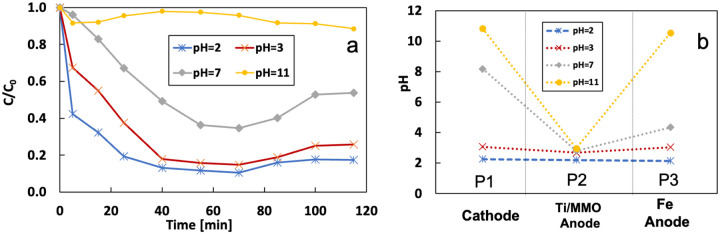
a) effect of the pH on the removal of IBP; b) pH value from different location of system at 60 mins

Figure 12a shows the effect of the pH on the removal of 3 mg/L of IBP. The removal rate was 82.7%, 74.3%, 46.3%, and 11.6% when the pH in the influent was 2, 3, 7, and 11, respectively. The removal rate is better under acidic conditions and decreases with the pH increase. Figure 12b shows the pH values from different sampling ports at 60 mins which do not vary significantly with time. pH value decreased at the anode and increased at the cathode due to the water electrolysis. Thus, the samples from P2 and P3 are acidic, and those from P1 are alkaline when the electrolyte is neutral. Water electrolysis is not the dominating reaction at the GF cathode, so pre-acidification can effectively control the pH value at the cathode. The pH from sampling P1 is slightly higher than the value from P2 and P3 when the initial pH is 2 and 3. Ti/MMO anode significantly reduces the alkaline electrolyte pH due to the strong water electrolysis reaction.

**Figure 13:**
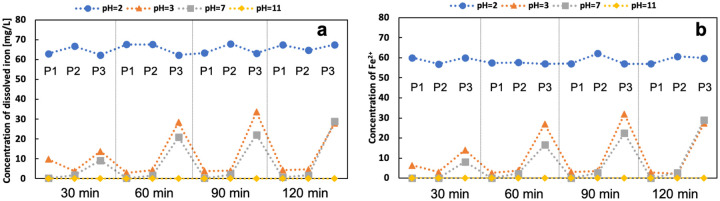
concentration of a) dissolved iron and b) Fe^2+^ under different pH of the electrolyte

pH value has a significant effect on the state of the iron in the electrolyte. Figure 13 shows the concentration of total dissolved iron and Fe^2+^ in the electrolyte at different sampling ports and times. The dissolved iron at the Fe anode (P3) is mainly ferrous. The concentration of total dissolved iron is slightly higher than Fe^2+^ due to the presence of Fe^3+^. This difference is not apparent because most of the Fe^3+^ precipitated. The concentration of dissolved iron decreases with the pH increase. There is minimal dissolved iron in the alkaline electrolyte. It is worth noting that the iron concentration is extraordinarily high when pH is 2 due to the iron dissolution at a very low pH. The Fenton reaction requires dissolved iron as the catalyst to produce OH radicals. Thus, the removal rate is high under pH of 2 and 3. Although the modified GF generated more H_2_O_2_ under alkaline conditions, the removal rate remained low without enough dissolved iron.

### 3.2. Degradation of ibuprofen by FeSO_4_ as the iron source

#### 3.2.1 Effect of the concentration of FeSO_4_

**Figure 14:**
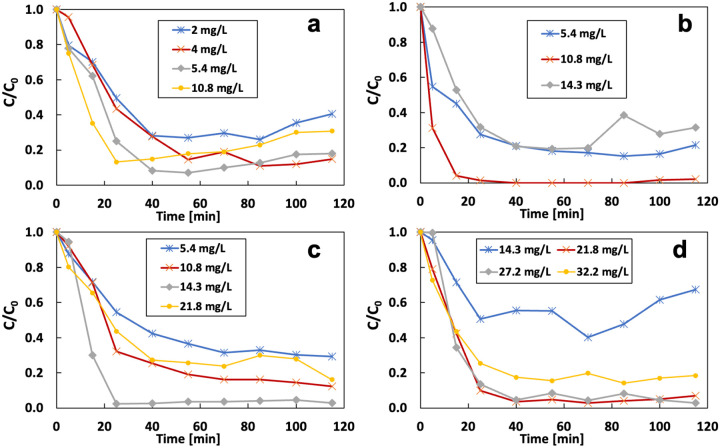
Effect of the Fe^2+^ concentration on the removal of IBP under different applied current: a) 60 mA; b) 90 mA; c) 120 mA; d) 200 mA

Figure 14 shows the effect of Fe^2+^ concentration and current on removing ibuprofen in the flow-through system. All experiments were conducted under the flow rate of 3 mL/min and the initial IBP of 1 mg/L. The removal of IBP dramatically decreased, which approximately followed the pseudo-first-order reaction kinetics, then reached the steady state. This significant decrease at the beginning is due to the strong Fenton reaction. Iron existed in the electrolyte in the form of Fe^2+^, and no precipitation was observed during this period. The optimum removal rate (86%) under 60 mA appears at the initial Fe^2+^ concentration of 4 mg/L (Figure 14a). Reducing the initial Fe^2+^ content to 2 mg/L reduces the degradation to 60%. Not all the H_2_O_2_ can be activated to OH radicals when Fe^2+^ is insufficient. Raising Fe^2+^ content to 5.4 mg/L and 10.9 mg/L lowers the removal rate to 80% and 70%. More Fe^2+^ supplies a stronger catalytic cycle of the Fenton reaction. However, excessive Fe2+ in the electrolyte can compete with IBP for ROS, such as OH radicals. We also observed that more iron precipitate was produced on the cathode when the Fe^2+^ concentration increased. The deposit might inhibit the generation of H_2_O_2_ at the cathode. Increasing the applied current can change the optimum Fe value for the removal rate. When the current was 90 mA, the optimum Fe^2+^ content changed to 10.8 mg/L with a removal rate of 98%, compared to 79% at 5.4 mg/L and 69% at 14.3 mg/L. The optimum Fe concentration continues to rise to 14.3 mg/L under 120 mA and 21.8 mg/L under 200 mA. The optimum removal rate under 60 mA is lower than the others because insufficient H_2_O_2_ production leads to a lower OH radical production.

Increasing current can enhance the reaction of water electrolysis and 2e-ORR to produce more anodic O_2_ and H_2_O_2_. However, O_2_ can oxidize Fe^2+^ to Fe^3+^, which competes with the Fenton reaction for Fe^2+^. At the same time, the residual H_2_O_2_ can consume OH radicals to generate HO2·, which exhibits a low oxidation power compared with OH radicals (Equation 10) and is relatively unreactive to organic matters(Bielski et al. 2009). Thus, with the increase of applied current, the concentration of Fe must increase to guarantee the optimal OH radicals’ production for the removal of IBP.

**Figure 15:**
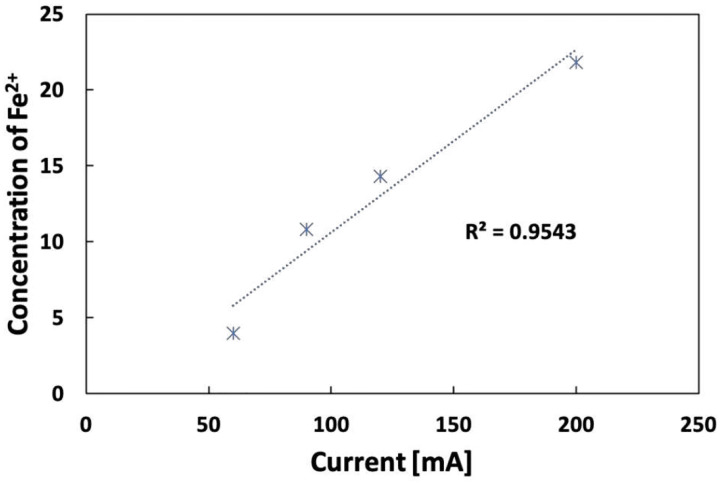
Correlation between initial Fe^2+^ content and current

The optimal Fe concentration for different currents (ranging from 60 mA to 200 mA) under 1 mg/L of IBP is summarized in Figure 15. A positive linear correlation exists between the optimum Fe content and the current. An equation to calculate the optimal Fe concentration (y, mg/L) under a specifically applied current, y=0.1202x-1.3993, was obtained. This equation can be used to direct the design of an EF/flow-through system.


Equation 8 $$H_{2}O_{2}+OH\cdot \to H_{2}O+HO_{2}\cdot$$

#### 3.2.2 Fe^2+^ regeneration on the modified cathode

**Figure 16:**
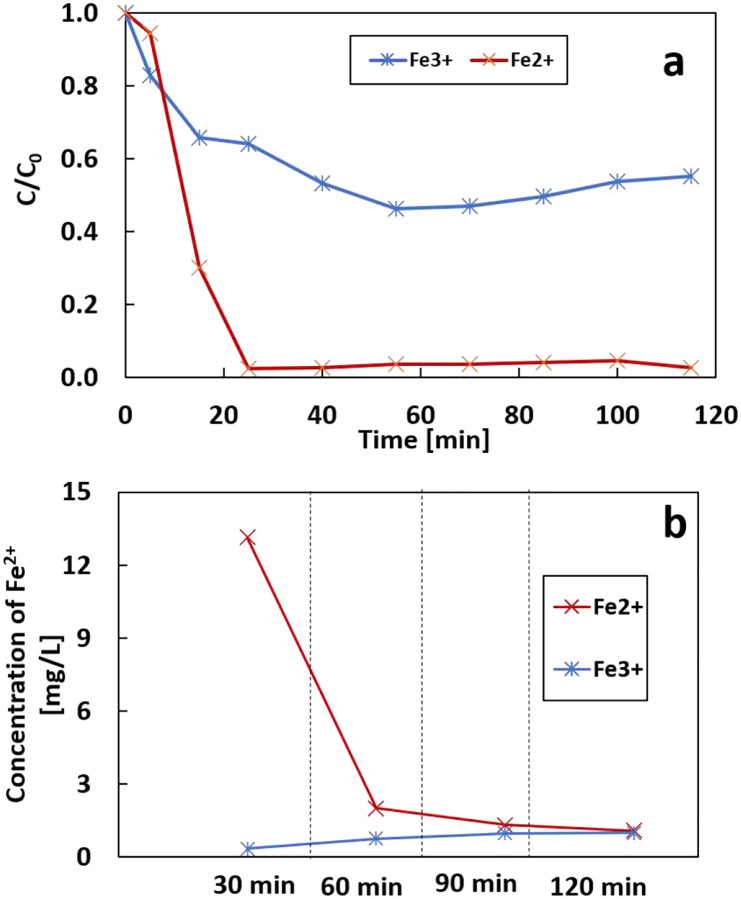
a) the removal rate of IBP by Fe^2+^ and Fe^3+^; b) the concentration of Fe^2+^ in the Fe^2+^-system and Fe^3+^-system

Fe_2_(SO_4_)_3_ was used as the iron source to test the regeneration of Fe^3+^ to Fe^2+^ at the cathode. The initial concentration of Fe^2+^ and Fe^3+^ are the same, with the removal rate of 97.3% and 45% at 115 mins, respectively (Figure 16a). The experiment with Fe^3+^ has a lower reaction rate than the Fe^2+^ because Fe^3+^ cannot directly activate the Fenton reaction. Figure 16b compares the concentration of Fe^2+^ above the cathode in two systems. The concentration of Fe^2+^ in the Fe^2+^-system decreases with time due to the consumption of Fe^2+^ by Fenton reaction and oxidants such as OH radicals. The presence of Fe^2+^ was also detected in Fe^3+^- system, and the concentration increased with time increase. This can be attributed to the reduction of Fe^3+^ on the graphite cathode. Consequently, this demonstrates that the modified GF cathode can be used for the regeneration of Fe^2+^ in the electro-Fenton process.

#### 3.2.3 Effect of the applied current

**Figure 17:**
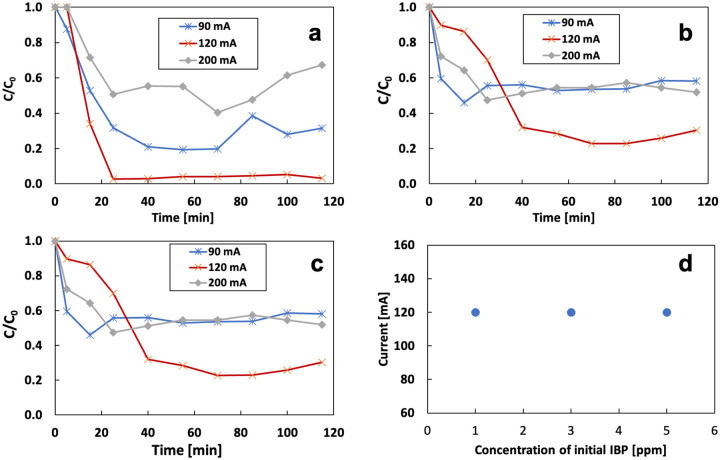
Effect of applied current on the removal rate with different initial IBP content under 120 mA: a) 1 mg/L; b) 3 mg/L; c) 5 mg/L; d) correlation between applied current and initial IBP content

Figure 17 shows the correlation between the current and the initial concentration of IBP on the removal rate with 14.3 mg/L of Fe^2+^. Figure 17a demonstrates the effect of current on the removal of 1 mg/L of IBP at the flow rate of 3 mL/min. The optimum removal rate is 97% at 115 mins with a current of 120 mA. Increasing the current to 200 mA or decreasing the current to 90 mA inhibited the removal rate to 33% and 60%, respectively. The same trend was observed at IBP concentration of 3 mg/L and 5 mg/L. The optimum removal rate appears at 120 mA under different initial ibuprofen content. This result matches the previous conclusion. Changing current can affect the electrochemical reactions in the system. As a stable contaminant, IBP does not react with H_2_O_2_ and O_2_ straightly. Thus, changing current cannot directly affect the removal rate. The optimum removal rate appears when the maximum production of OH radicals is achieved. IBP does not affect the OH radical generation (Figure 17d).

**Figure 18:**
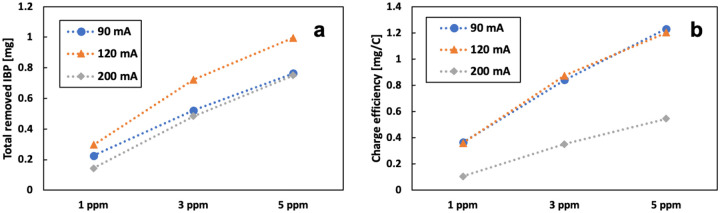
a) total removed IBP and b) charge efficiency under different current and initial concentration of IBP

Figure 18 shows the totally removed IBP from the system in 115 mins and the charge efficiency under different current conditions. The total removed IBP increased with increasing of the initial IBP. OH radicals were more inclined to react with IBP when the concentration of IBP in the electrolyte was increasing. The removal rate was low for the high initial concentration, but the quantity (in mass) of removal was high. The experiment under 120 mA current had the highest total removal because of the optimum production of OH radicals with 14.3 mg/L of Fe^2+^. Experiments at 90 mA and 120 mA had similar charge efficiency, both being higher than results at 200 mA, where excessive current can lead to H_2_O_2_ decomposition. A high concentration of H_2_O_2_ can compete with contaminants for OH radicals.

#### 3.2.4 Effect of the initial concentration of ibuprofen

**Figure 19:**
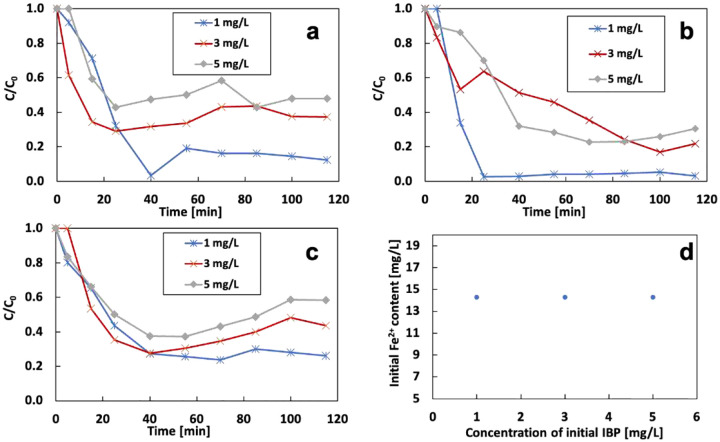
Effect of Fe^2+^ content in the electrolyte

Figure 19 analysis the correlation between iron content and initial concentration of IBP. The removal rate at 10.8 mg/L of Fe^2+^ is 87.5%, 62.7%, and 52.2% at initial IBP of 1, 3, and 5 mg/L. The optimum removal rate appears under 1 mg/L of IBP. Increasing current does not change this trend. The removal rate decreases with the increase of the initial concentration of IBP at the fixed Fe content. The lower removal rate of IBP at high concentration is attributed to the generation of degradation by-products that compete with the targeted contaminants for OH radicals. The previous study shows that the degradation of IBP by the EF process is mainly because of the destruction of the propionic acid group to produce 4-isobutylacetophenone(Yuan, Gou, et al. 2013). OH radicals prefer to attack 4-isobutylacetophenone because of the stronger conjugated effect from the carboxyl group and benzene. Therefore, the higher initial concentration can produce more by-products than the lower one, which will compete with the removal of IBP. The highest removal rate appears at 14.3 mg/L of Fe^2+^, which is the optimum Fe^2+^ value under 120 mA. There is no correlation between the iron content and the concentration of initial IBP.

#### 3.2.4 Effect of the pH of the electrolyte

**Figure 20:**
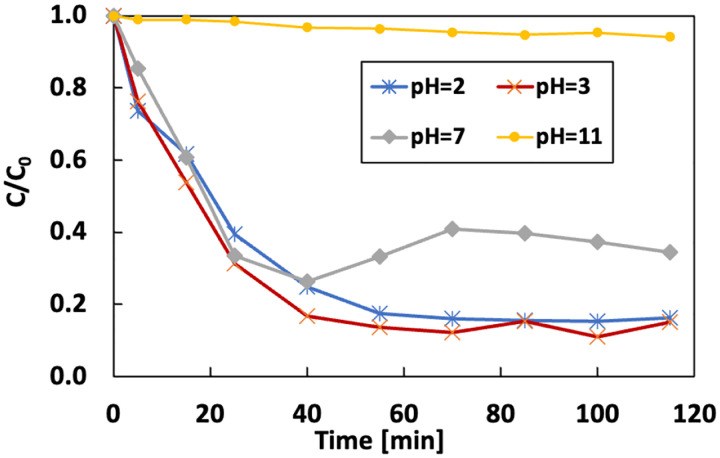
Effect of the pH on the removal of IBP

The effect of pH on the removal of IBP was evaluated under the initial IBP of 5 ppm and Fe content of 14.3 mg/L. The removal rate was 85% and 84% when the initial pH was adjusted to 2 and 3, respectively (Figure 20). Increasing pH to neutral and 11 reduced the removal rate to 66% and 6%.

**Figure 21:**
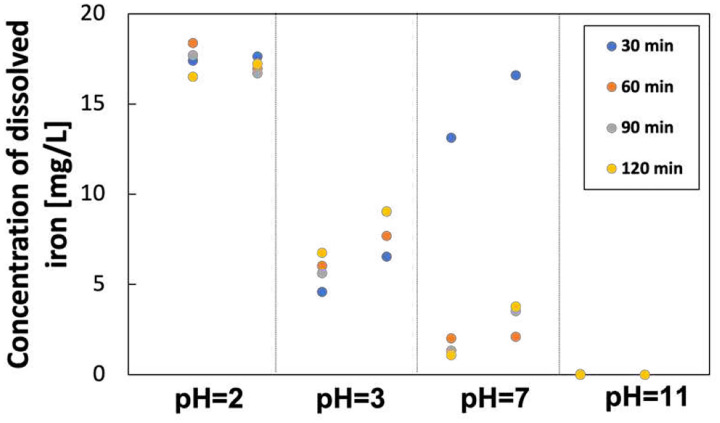
Variation of dissolved iron concentration with time at different pH

Figure 21 shows the variation of dissolved iron with time at different pH of the electrolyte. Under acidic conditions, the total dissolved iron did not fluctuate much with time. There was a significant drop under pH 7 after 30 mins due to the pH change around the electrode. As the pH stabilized with time, the concentration of the dissolved iron became stable. Iron concentration in pH 11 is below the detection limit for the spectrophotometer because of the formation of iron precipitation. Classic Fenton uses soluble iron (Fe^2+^ or Fe^3+^ form) to guarantee the highest process efficiency. Fe^2+^ can be quickly oxidized to Fe^3+^, and most of the Fe^3+^ will produce ferric hydroxide sludge when the pH is greater than 4. Thus, the optimum pH for the Fenton reaction is between 2.8 to 3. Although the previous study showed that the modified GF has better performance under alkaline conditions, the Fenton reaction can be greatly inhibited. Under the optimum pH, more H2O2 can be activated to OH radicals for the degradation reaction.

#### 3.2.5 Effect of the flow rate

**Figure 22:**
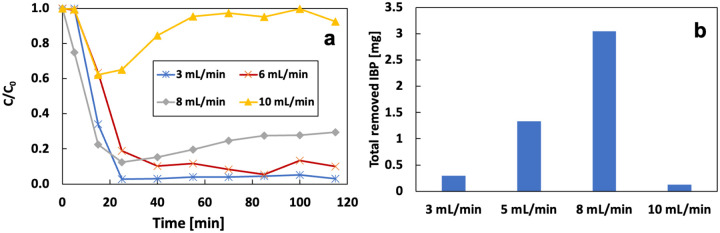
a) effect of the flow rate on the removal of 1 mg/L of IBP under 120 mA and 14.2 mg/L of initial Fe^2+^; b) total removal IBP under different flow rate

Figure 22 shows the effect of flow rate on the removal of IBP in the flow-through system. All experiments were conducted under 120 mA with the initial IBP of 1 mg/L and Fe of 14.3 mg/L. The removal of IBP was 97%, 91%, 89%, and 8% at flow rates of 3, 6, 8, and 10 mL/min at 115 mins. Increasing the flow rate can dilute the concentration of H2O2 and OH radicals, but the concentration of IBP and Fe^2+^ remains the same. Thus, increasing the flow rate can decrease the removal rate. The total removed IBP from the system in 115 mins increases with the flow rate increase from 3 to 8 mL/min then decreases when the flow rate keeps increasing. The concentration of OH radicals is sufficient to react with most of the IBP in the system when the flow rate is less than or equal to 8 mL/min. Because the high flow rate will carry more IBP through the system at the same period, the high flow rate has a high value of total removed IBP. However, when the flow rate keeps increasing to 10 mL/min, the concentration of H2O2 is too low to generate enough OH radicals for the system. The removal rate and the total removed IBP are then very low. The FeSO4 system is less sensitive to the flow rate compared to the iron anode system.

## 4. Conclusion

This research evaluated the performance of two iron sources (cast iron anode and FeSO_4_ salt) on the removal of ibuprofen by electro-Fenton process in the flow-through system. Experimental results indicate that cast iron anode located at the bottom of the electrode’s sequence achieve the best performance for the ibuprofen removal. The optimum split current on iron anode is 3 mA when the total current is 120 mA. Lower split current cannot generate sufficient Fe^2+^ for the Fenton process but higher split current can increase the iron precipitation. There is a linear correlation between the applied current and the concentration of the concentration of Fe^2+^ in the FeSO_4_-system. However, there is no correlation between concentration of Fe^2+^ and IBP concentration or current and initial IBP concentration. Both systems have an optimum applied current at which the accumulated H_2_O_2_ concentration is maximum. Increasing flow rate can decrease the IBP removal rate due to the decreasing of the oxygen retention time for the H_2_O_2_ production and the dilution of H_2_O_2_ concentration. However, compared to FeSO_4_-system, flow rate has more effect on cast iron system because it dilutes the Fe^2+^ concentration as well. Both systems prefer to operate under acidic conditions because the Fe^2+^ will be less susceptible to be generate iron precipitation. Cast iron works as an external Fe^2+^ for the electro-Fenton process, which can be used for the iron-free water conditions or the water without enough Fe^2+^. FeSO_4_ has better resistance to the flow rate changing. It can be used for the high flow rate system. These findings contribute in several ways to our understanding of the electro-Fenton process under flow conditions and provide a basis for how to design the reactor for the water treatment.

## Figures and Tables

**Table 1:** The pseudo-first-constant value, the time required to reach the steady state, the delay time and correlation coefficient at different applied current

Current (mA)	Time (min)	Delay (min)	R^2^ value	K value
60	70	5	95.3%	0.0036
90	55	5	93.9%	0.013
120	55	0	99.7%	0.0199
150	55	0	93.2%	0.0106

**Table 2:** The pseudo-first-constant value, the time required to reach the steady state, the delay time and correlation coefficient at flow rate

Flow rate (mL/min)	Time (min)	Delay (min)	R^2^ value	K value
3	70	5	98.6%	0.0364
5	55	5	99.5%	0.0145
7	40	5	98.5%	0.0046

## Data Availability

The data sets used or analyzed in the current study are available from the corresponding author upon reasonable request.

## References

[R1] Ai Zhihui, Xiao Haiyan, Mei Tao, Liu Juan, Zhang Lizhi, Deng Kejian, and Qiu Jianrong. 2008. “Electro-Fenton Degradation of Rhodamine B Based on a Composite Cathode of Cu2O Nanocubes and Carbon Nanotubes.” Journal of Physical Chemistry C 112 (31): 11929–35. 10.1021/JP803243T/SUPPL_FILE/JP803243T-FILE002.PDF.

[R2] Bielski Benon H.J., Cabelli Diane E., Arudi Ravindra L., and Ross Alberta B.. 2009. “Reactivity of HO2/O−2 Radicals in Aqueous Solution.” Journal of Physical and Chemical Reference Data 14 (4): 1041. 10.1063/1.555739.

[R3] Brillas Enric, and Casado Juan. 2002. “Aniline Degradation by Electro-Fenton^®^ and Peroxi-Coagulation Processes Using a Flow Reactor for Wastewater Treatment.” Chemosphere 47 (3): 241–48. 10.1016/S0045-6535(01)00221-1. 11996144

[R4] Brillas Enric, Sire Ignasi, Oturan Mehmet A, Sirés Ignasi, and Oturan Mehmet A. 2009. “Electro-Fenton Process and Related Electrochemical Technologies Based on Fenton ‘s Reaction Chemistry.” Chemical Reviews 109 (12): 6570–6631. 10.1021/cr900136g. 19839579

[R5] Brillas Enric, Sirés Ignasi, and Oturan Mehmet A.. 2009. “Electro-Fenton Process and Related Electrochemical Technologies Based on Fenton’s Reaction Chemistry.” Chemical Reviews 109 (12): 6570–6631. 10.1021/cr900136g. 19839579

[R6] Castañeda Locksley F., Walsh Frank C., Nava José L., and Ponce de León Carlos. 2017. “Graphite Felt as a Versatile Electrode Material: Properties, Reaction Environment, Performance and Applications.” Electrochimica Acta 258 (December): 1115–39. 10.1016/J.ELECTACTA.2017.11.165.

[R7] Cretin Marc, and Oturan Mehmet A.. 2016. Electro-Fenton Process. Encyclopedia of Membranes. 10.1007/978-3-662-44324-8_2043.

[R8] Festos H J H., By n.d. “LXXIII.—Oxidation of Tartaric Acid in Presence of Iron,” 899–910.

[R9] Gao Guandao, Zhang Qiaoying, Hao Zhenwei, and Vecitis Chad D.. 2015. “Carbon Nanotube Membrane Stack for Flow-through Sequential Regenerative Electro-Fenton.” Environmental Science and Technology 49 (4): 2375–83. 10.1021/es505679e. 25602741

[R10] Haber F, Weiss J, Seph J O, and Eiss W. 1934. “The Catalytic Decomposition of Hydrogen Peroxide by Iron Salts.” Proceedings of the Royal Society of London. Series A - Mathematical and Physical Sciences 147 (861): 332–51. 10.1098/RSPA.1934.0221.

[R11] Jones Adele M., Griffin Phillipa J., Collins Richard N., and Waite T. David. 2014. “Ferrous Iron Oxidation under Acidic Conditions – The Effect of Ferric Oxide Surfaces.” Geochimica et Cosmochimica Acta 145 (November): 1–12. 10.1016/J.GCA.2014.09.020.

[R12] Khataee A R, Safarpour M, Zarei M, and Aber S. 2011. “Electrochemical Generation of H2O2 Using Immobilized Carbon Nanotubes on Graphite Electrode Fed with Air: Investigation of Operational Parameters.” Journal of Electroanalytical Chemistry 659 (1): 63–68.

[R13] Kim Hyo Won, Ross Michael B., Kornienko Nikolay, Zhang Liang, Guo Jinghua, Yang Peidong, and McCloskey Bryan D.. 2018. “Efficient Hydrogen Peroxide Generation Using Reduced Graphene Oxide-Based Oxygen Reduction Electrocatalysts.” Nature Catalysis 2018 1:4 1 (4): 282–90. 10.1038/s41929-018-0044-2.

[R14] Komadel Peter, and Stucki Joseph W.. 1988. “Quantitative Assay of Minerals for Fe2+ and Fe3+ Using 1,10-Phenanthroline: III. A Rapid Photochemical Method.” Clays and Clay Minerals 1988 36:4 36 (4): 379–81. 10.1346/CCMN.1988.0360415.

[R15] Le Thi Xuan Huong, Bechelany Mikhael, Lacour Stella, Oturan Nihal, Oturan Mehmet A., and Cretin Marc. 2015. “High Removal Efficiency of Dye Pollutants by Electron-Fenton Process Using a Graphene Based Cathode.” Carbon 94 (November): 1003–11. 10.1016/J.CARBON.2015.07.086.

[R16] Lin Heng, Zhang Hui, Wang Xue, Wang Liguo, and Wu Jie. 2014. “Electro-Fenton Removal of Orange II in a Divided Cell: Reaction Mechanism, Degradation Pathway and Toxicity Evolution.” Separation and Purification Technology 122 (February): 533–40. 10.1016/J.SEPPUR.2013.12.010.

[R17] Liu R. J., Crozier P. A., Smith C. M., Hucul D. A., Blackson J., and Salaita G.. 2005. “Metal Sintering Mechanisms and Regeneration of Palladium/Alumina Hydrogenation Catalysts.” Applied Catalysis A: General 282 (1–2): 111–21. 10.1016/j.apcata.2004.12.015.

[R18] Mao Xuhui, Ciblak Ali, Baek Kitae, Amiri Mohammad, Loch-Caruso Rita, and Alshawabkeh Akram N.. 2012. “Optimization of Electrochemical Dechlorination of Trichloroethylene in Reducing Electrolytes.” Water Research 46 (6): 1847–57. 10.1016/j.watres.2012.01.002. 22264798 PMC3288245

[R19] Mao Xuhui, Yuan Songhu, Fallahpour Noushin, Ciblak Ali, Howard Joniqua, Padilla Ingrid, Loch-Caruso Rita, and Alshawabkeh Akram N.. 2012. “Electrochemically Induced Dual Reactive Barriers for Transformation of TCE and Mixture of Contaminants in Groundwater.” Environmental Science and Technology 46 (21): 12003–11. 10.1021/es301711a 23067023 PMC3493133

[R20] Belosevic Miodrag, El Din Mohamed Gamal Zengquan Shu Bolton James R.. 2014. “Degradation of Alizarin Yellow R Using UV / H 2 O 2 Advanced Oxidation Process.” Environmental Science & Technology 33 (2): 482–89. 10.1002/ep.

[R21] Oturan Mehmet A., Guivarch Elodie, Oturan Nihal, and Sirés Ignasi. 2008. “Oxidation Pathways of Malachite Green by Fe3+-Catalyzed Electro-Fenton Process.” Applied Catalysis B: Environmental 82 (3–4): 244–54. 10.1016/J.APCATB.2008.01.016.

[R22] Sheng Yiping, Song Shili, Wang Xiuli, Song Laizhou, Wang Chunjia, Sun Honghong, and Niu Xueqing. 2011. “Electrogeneration of Hydrogen Peroxide on a Novel Highly Effective Acetylene Black-PTFE Cathode with PTFE Film.” Electrochimica Acta 56 (24): 8651–56. 10.1016/J.ELECTACTA.2011.07.069.

[R23] Sheng Yiping, Zhao Yue, Wang Xiuli, Wang Rui, and Tang Ting. 2014. “Electrogeneration of H2O2 on a Composite Acetylene Black-PTFE Cathode Consisting of a Sheet Active Core and a Dampproof Coating.” Electrochimica Acta 133: 414–21. 10.1016/j.electacta.2014.04.071.

[R24] Sirés Ignasi, Garrido José Antonio, Rodríguez Rosa María, Brillas Enric, Oturan Nihal, and Oturan Mehmet A.. 2007. “Catalytic Behavior of the Fe3+/Fe2+ System in the Electro-Fenton Degradation of the Antimicrobial Chlorophene.” Applied Catalysis B: Environmental 72 (3–4): 382–94. 10.1016/J.APCATB.2006.11.016.

[R25] Su Pei, Zhou Minghua, Lu Xiaoye, Yang Weilu, Ren Gengbo, and Cai Jingju. 2019. “Electrochemical Catalytic Mechanism of N-Doped Graphene for Enhanced H2O2 Yield and in-Situ Degradation of Organic Pollutant.” Applied Catalysis B: Environmental 245 (May): 583–95. 10.1016/J.APCATB.2018.12.075.

[R26] Yamanaka Ichiro, Onizawa Takeshi, Takenaka Sakae, and Otsuka Kiyoshi. 2003. “Direct and Continuous Production of Hydrogen Peroxide with 93% Selectivity Using a Fuel-Cell System.” Angewandte Chemie - International Edition 42 (31): 3653–55. 10.1002/anie.200351343. 12916038

[R27] Yu Fangke, Zhou Minghua, and Yu Xinmin. 2015. “Cost-Effective Electro-Fenton Using Modified Graphite Felt That Dramatically Enhanced on H2O2 Electro-Generation without External Aeration.” Electrochimica Acta 163: 182–89. 10.1016/j.electacta.2015.02.166.

[R28] Yu Fangke, Zhou Minghua, Zhou Lei, and Peng Rudan. 2014. “A Novel Electro-Fenton Process with H2O2 Generation in a Rotating Disk Reactor for Organic Pollutant Degradation.” Environmental Science and Technology Letters. 10.1021/ez500178p.

[R29] Yu Xinmin, Zhou Minghua, Ren Gengbo, and Ma Liang. 2015. “A Novel Dual Gas Diffusion Electrodes System for Efficient Hydrogen Peroxide Generation Used in Electro-Fenton.” Chemical Engineering Journal 263: 92–100. 10.1016/j.cej.2014.11.053.

[R30] Yuan Songhu, Chen Mingjie, Mao Xuhui, and Alshawabkeh Akram N.. 2013. “A Three-Electrode Column for Pd-Catalytic Oxidation of TCE in Groundwater with Automatic PH-Regulation and Resistance to Reduced Sulfur Compound Foiling.” Water Research 47 (1): 269–78. 10.1016/j.watres.2012.10.009. 23121896 PMC3581803

[R31] Yuan Songhu, Gou Na, Alshawabkeh Akram N., and Gu April Z.. 2013. “Efficient Degradation of Contaminants of Emerging Concerns by a New Electro-Fenton Process with Ti/MMO Cathode.” Chemosphere 93 (11): 2796–2804. 10.1016/j.chemosphere.2013.09.051. 24125716

[R32] Yuan Songhu, Tian Meng, Cui Yanping, Lin Li, and Lu Xiaohua. 2006. “Treatment of Nitrophenols by Cathode Reduction and Electro-Fenton Methods.” Journal of Hazardous Materials 137 (1): 573–80. 10.1016/J.JHAZMAT.2006.02.069. 16650528

[R33] Zhang Guoquan, Zhou Yufei, and Yang Fenglin. 2015. “FeOOH-Catalyzed Heterogeneous Electro-Fenton System upon Anthraquinone@Graphene Nanohybrid Cathode in a Divided Electrolytic Cell: Catholyte-Regulated Catalytic Oxidation Performance and Mechanism.” Journal of The Electrochemical Society 162 (6): H357–65. 10.1149/2.0691506JES/XML.

[R34] Zhang Huichun, and Lemley Ann T.. 2006. “Reaction Mechanism and Kinetic Modeling of DEET Degradation by Flow-through Anodic Fenton Treatment (FAFT).” Environmental Science and Technology 40 (14): 4488–94. 10.1021/es060515b. 16903290

[R35] Zhang Xingwang, Lei Lecheng, Xia Bing, Zhang Yi, and Fu Jianliang. 2009. “Oxidization of Carbon Nanotubes through Hydroxyl Radical Induced by Pulsed O 2 Plasma and Its Application for O 2 Reduction in Electro-Fenton.” Electrochimica Acta 54: 2810–17. 10.1016/j.electacta.2008.11.029.

[R36] Zhao Yuwei, Cui Jiaxin, Zhou Wei, Hojabri Shayan, and Alshawabkeh Akram N.. 2020. “Electrogeneration of H2O2 Utilizing Anodic O2 on a Polytetrafluoroethylene-Modified Cathode in a Flow-through Reactor.” Electrochemistry Communications 121: 106868. 10.1016/j.elecom.2020.106868. 33981182 PMC8112623

[R37] Zhao Yuwei, Hojabri Shayan, Sarrouf Stephanie, and Alshawabkeh Akram N.. 2022. “Electrogeneration of H2O2 by Graphite Felt Double Coated with Polytetrafluoroethylene and Polydimethylsiloxane.” Journal of Environmental Chemical Engineering 10 (4): 108024. 10.1016/j.jece.2022.108024. 36969726 PMC10035042

[R38] Zhou Minghua, Yu Qinghong, Lei Lecheng, and Barton Geoff. 2007. “Electro-Fenton Method for the Removal of Methyl Red in an Efficient Electrochemical System.” Separation and Purification Technology 57 (2): 380–87. 10.1016/j.seppur.2007.04.021.

[R39] Zhou Wei, Meng Xiaoxiao, Gao Jihui, and Alshawabkeh Akram N.. 2019. “Hydrogen Peroxide Generation from O2 Electroreduction for Environmental Remediation: A State-of-the-Art Review.” Chemosphere 225: 588–607. 10.1016/j.chemosphere.2019.03.042. 30903840 PMC6921702

[R40] Zhou Wei, Rajic Ljiljana, Meng Xiaoxiao, Nazari Roya, Zhao Yuwei, Wang Yan, Gao Jihui, Qin Yukun, and Alshawabkeh Akram N.. 2019. “Efficient H2O2 Electrogeneration at Graphite Felt Modified via Electrode Polarity Reversal: Utilization for Organic Pollutants Degradation.” Chemical Engineering Journal 364 (September 2018): 428–39. 10.1016/j.cej.2019.01.175. 32581640 PMC7314056

[R41] Zhou Wei, Rajic Ljiljana, Zhao Yuwei, Gao Jihui, Qin Yukun, and Alshawabkeh Akram N.. 2018. “Rates of H2O2 Electrogeneration by Reduction of Anodic O2 at RVC Foam Cathodes in Batch and Flow-through Cells.” Electrochimica Acta 277: 185–96. 10.1016/j.electacta.2018.04.174. 32153302 PMC7062376

